# Introgression of the *RppQ* gene from field corn improves southern rust resistance in sweet corn

**DOI:** 10.1007/s11032-022-01315-7

**Published:** 2022-08-30

**Authors:** Nan Zhang, Xitao Qi, Xiaofeng Li, Guangyu Li, Gaoke Li, Jianguang Hu

**Affiliations:** Crops Research Institute, Guangdong Academy of Agricultural Sciences, Guangdong Key Laboratory of Crops Genetics and Improvement, Guangzhou, 510640 China

**Keywords:** Sweet corn, Southern rust resistance, Qi319, *RppQ*, Marker-assisted selection

## Abstract

Southern rust, one of the most destructive foliar diseases of sweet corn (*Zea mays* convar. *saccharata* var. *rugosa*), is caused by *Puccinia polysora* Underw. and leads to enormous yield losses and reduced quality of sweet corn in China. Utilization of resistance genes is an effective and environmentally friendly strategy for improving southern rust resistance of sweet corn. However, improvement is hampered by a lack of resistance genes in Chinese sweet corn germplasm. In this study, we introgress the southern rust resistance gene *RppQ* from Qi319, an inbred line of southern rust–resistant field corn, into four elite sweet corn inbred lines (1401, 1413, 1434, and 1445) using marker-assisted backcross breeding. These are parental inbred lines of four popular sweet corn varieties: Yuetian 28, Yuetian 13, Yuetian 26, and Yuetian 27. We developed five *RppQ*-based markers (M0607, M0801, M0903, M3301, and M3402) and employed these markers for foreground selection; 92.3 to 97.9% of the recurrent parent genomes were recovered following three or four rounds of backcrossing. The four newly developed sweet corn lines all showed significant improvement of southern rust resistance compared with their respective parent lines. Meanwhile, there was no significant difference in phenotypic data for agronomic traits. In addition, reconstituted hybrids derived from the converted lines retained resistance to southern rust, while other agronomic traits and sugar content remained unchanged. Our study provides an example of successful development of southern rust–resistant sweet corn using a resistance gene from field corn.

**Supplementary Information:**

The online version contains supplementary material available at 10.1007/s11032-022-01315-7.

## Introduction

Sweet corn (*Zea mays* convar. *saccharata* var. *rugosa*) is a type of specialty corn carrying one or more mutations in the endosperm starch biosynthesis pathway that result in increased sugar content and altered polysaccharide composition (Revilla et al. 2021). Sweet corn is eaten at the immature stage as a fruit or vegetable and provides an important source of fiber, minerals, and vitamins (Lertrat et al. 2007). Its good taste and high nutrient content have driven fast expansion of the sweet corn market worldwide. Mutations in three genes are widely used in sweet corn breeding: (1) *sugary1* (*su1*), encoding a starch debranching enzyme (Pan et al. 1984; James et al. 1995); (2) *sugary enhancer1* (*se1*), a recessive modifier of *sugary1* (Zhang et al. 2019); and (3) *shrunken2* (*sh2*), encoding the large subunit of ADP-glucose pyrophosphorylase (AGPase) (Hannah et al. 1976). Sweet corn breeding started in the 1950s in China, and a series of sweet corn materials was introduced from the USA in the 1960s (Yao et al. 2011). The first hybrid, ‘Baishatang Sweet,’ a *su1* variety, was released in 1968 (Yao et al. 2011). In the 1980s and 1990s, farmers in southeast China were employed to grow sweet corn for fresh ear export by business people from Hong Kong or Taiwan. The area used for sweet corn production has dramatically increased in recent decades to coincide with the marked growth of sweet corn consumption in Mainland China, with sweet corn becoming one of the major cash crops for farmers in South China.

Sweet corn production faces threats from diverse diseases, with one of the major challenges being southern rust, an airborne disease caused by the fungus *Puccinia polysora* Underw. (Ramirez‐Cabral et al. 2017; Debnath et al. 2019). This disease occurs globally, especially under warm humid conditions, leading to significant yield losses. Spores are found first on the upper surface of the lower leaves of infected plants, then advance upwards and spread to neighboring plants. Orange to tan, circular or oval pustules form on the upper leaf surface. Secondary infection continues under favorable conditions, and repeated cycles quickly result in epidemic levels, causing leaf defoliation and premature senescence (Debnath et al. 2019). In severe cases, the leafy husks are covered with orange spores, making the ears of sweet corn unsuitable for sale.

Southern rust was first reported in 1972 in Ledong, Hainan Province, China, and has been recorded in more than 20 provinces/cities in the past 50 years (Duan et al. 1984; Sun et al. 2021). Disease areas are divided into overwinter regions, where maize is planted throughout the year, guaranteeing the overwintering and survival of *P. polysora*, and epidemic regions, where wind transports urediniospores from different origins to the major summer maize-producing area (Sun et al. 2021; Wang et al. 2020a). A double cropping system is applied for growing sweet corn in Guangdong Province. The first crop is planted in the spring season, and the second crop is planted in the autumn season, enabling winter survival of the pathogen and making southern rust an annual disease in November.

Chemical control of *P. polysora* has side effects for the environment and can cause public concerns over food safety. Deployment of disease-resistant varieties is therefore one of the most economical and sustainable means for combating southern rust. Genetic research on resistance to southern rust spans decades, and several resistance genes for southern rust have been mapped (Brewbaker et al. 2011; Sun et al. 2021). As early as 1957, *Rpp1* and *Rpp2*, conferring resistance to different races of *P. polysora*, EA1 and EA2, were identified from maize lines in Colombia and Mexico, respectively (Storey et al. 1957). Subsequently, *Rpp3*–*Rpp8* were identified in different maize lines (Robert et al. 1962). In 1965, Ullstrup (1965) reported on the resistance gene *Rpp9*, with resistance to race PP.9. Two other race-specific resistance genes, *Rpp10* and *Rpp11*, exhibiting resistance to EA1 and EA3, were identified afterwards (Storey et al. 1967). Maize inbred line Qi319 was reported to carry the resistance gene *RppQ* (Chen et al. 2004; Zhou et al. 2007), and *RppD* was mapped using an F_2_ and F_2:3_ group derived from the resistant line W2D and the susceptible line W222 (Zhang et al. 2010). Zhao et al. (2013) used a pair of near isogenic lines, F939 and F349, to locate the disease resistance gene *Rpp25*, while *RppS* was identified from a tropical inbred line, SCML205 (Wu et al. 2015). Wang et al. (2020b) identified and mapped the southern corn rust resistance gene *RppM* from the near-isogenic line Kangxiujing2416 (Jing2416K). Lv et al. (2021) detected the major quantitative trait locus (QTL) *RppCML496* in the tropical resistant line CML496 and finally identified the resistance gene as *RppC* (Deng et al. 2022). The abovementioned disease-resistant genes (*RppQ*, *RppD*, *Rpp25*, *RppS*, *RppM*, and *RppCML496*) are all located on the short arm of chromosome 10, suggesting that the nucleotide binding site leucine-rich repeat (NBS-LRR) protein family cluster in this region plays a role in resistance to different physiological races (Wu et al. 2015). Other novel QTLs conferring southern rust resistance have also been identified, including several QTLs from tropical sweet corn (Wanlayaporn et al. 2013; Deng et al. 2019; Lu et al. 2020). The rapid development of bioinformatics has made molecular breeding very convenient, and a combination of conventional and molecular breeding can achieve precise and fast introgression of resistance genes. The field corn inbred line Qi319, an elite Chinese field corn germplasm carrying *RppQ*, has displayed durable southern rust resistance for decades, making it an ideal donor for improvement of southern rust resistance in sweet corn (Chen et al. 2004; Zhou et al. 2007).

The sweet corn breeding program in China has focused on yield and sweetness and produced many popular cultivars such as “Yuetian 13,” “Yuetian 26,” “Yuetian 27,” and “Yuetian 28.” Most of these cultivars are susceptible or highly susceptible to southern rust. Although several studies on southern rust resistance genes or loci have been reported, few studies have attempted to decipher southern rust resistance genetics in sweet corn, making it difficult to utilize resistance resources from sweet corn directly. *RppQ*-linked markers have been developed during the mapping process; however, these markers are not completely suitable for all inbred lines with diverse genetic backgrounds. Therefore, we developed five new molecular markers in the *RppQ* mapping region and introgressed the Qi319 disease resistance section into four elite sweet corn inbred lines (1401, 1413, 1434, and 1445) to obtain enhanced southern rust resistance in sweet corn.

## Materials and methods

### Plant materials

The field corn inbred line Qi319, derived from the US maize hybrid 78,599 and carrying *RppQ*, was used as the donor parent (Chen et al. 2004; Lu et al. 2020; Zhou et al. 2007). Four *sh2* elite sweet corn lines, 1401, 1413, 1434, and 1445, were used as recipient and recurrent parents. These four lines were developed by breeders from the Crops Research Institute, Guangdong Academy of Agricultural Sciences, and are the parental lines of four popular commercial hybrids, namely ‘Yuetian13’ (1413 × 13F), ‘Yuetian26’ (1445 × 26F), ‘Yuetian 27’ (1434 × 27F), and ‘Yuetian 28’ (1401 × 28F). They possess excellent eating quality and high yield, but are susceptible or highly susceptible to southern rust. The study was carried out from 2016 to 2020 at Zhongluotan experiment station in Guangdong Province during both the spring and autumn growing seasons. Recommended agronomic management practices were adopted to raise the plants.

### Isolation of genomic DNA and genotyping

For PCR-based genotyping of functional DNA markers, leaf samples were collected from the field 4 weeks after sowing. Genomic DNA was extracted from leaves using the CTAB method (Murray et al. 1980) and subsequently quantified using a NanoDrop spectrophotometer. PCR was performed in 10 μl volume using 5 μl PCR Mix (Trans Gene, China), 1 μl genomic DNA (100 ng/μl), 0.2 μl each forward and reverse primer (10 μM), and 3.6 μl ddH_2_O. The PCR program was as follows: initial denaturation at 95 °C for 30 s, 35 cycles of denaturation at 95 °C for 30 s, annealing at 60 °C for 60 s, extension at 72 °C for 60 s, and a final extension at 72 °C for 10 min. PCR products were separated on 2% (w/v) agarose gel, stained with ethidium bromide, and visualized under UV light for scoring. A 2000-bp DNA ladder (Trans Gene, China) was used as a reference for amplified fragments.

### Primer design and foreground selection

The nucleotide sequence of the *RppQ* mapping region was download from the B73 release genomic annotation database (https://www.maizegdb.org/) (Chen et al. 2004; Zhou et al. 2007) and aligned with the whole genomic sequence. Single- or low-copy sequences were selected for designing markers using Primer 3 (http://bioinfo.ut.ee/primer3-0.4.0/primer3/). Amplification products were aligned with the B73 reference sequence to ensure primers were correctly targeted. Markers showing polymorphism between the donor and recipient parent lines on 2% (w/v) agarose gel were designated as InDel markers, and both dominant and co-dominant markers were selected. Corresponding polymorphic markers were used for foreground selection in each of the backcross and selfed generations for selecting individuals carrying *RppQ*.

### Generation of marker-assisted backcross populations

**Fig. 1:**
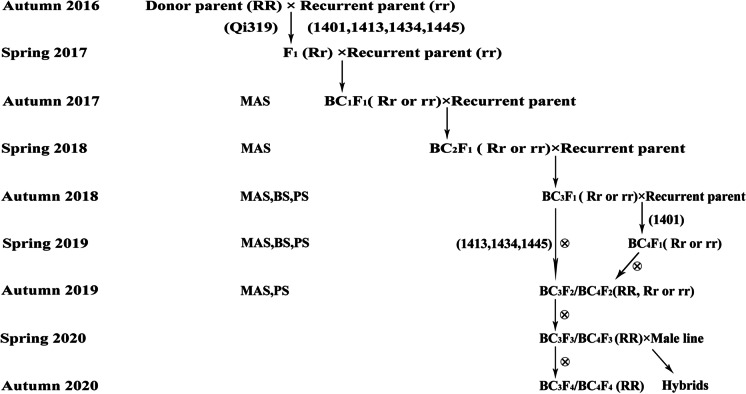
Marker-assisted breeding scheme for introducing the *RppQ* gene from field corn inbred line Qi319 into four sweet corn lines. *RR* homozygous allele from donor parent, *rr* homozygous allele from recurrent parent, *Rr* heterozygous allele, *MAS* marker-assisted selection, *BS* background selection, *PS* phenotype selection

For target introgression of *RppQ* into the four sweet corn inbred lines, a stepwise transfer marker–assisted backcross breeding program was adopted (Fig. 1). The donor, inbred Qi319, was crossed with four sweet corn lines to develop F_1_ hybrids, respectively. F_1_ hybrids were grown during the spring season of 2017, and their hybridity was tested using co-dominant markers for *RppQ*. Confirmed F_1_ hybrids were backcrossed with the respective susceptible lines to generate BC_1_F_1_ lines. BC_1_F_1_ lines from the four crosses were grown during the autumn season of 2017. Individuals were examined using the respective markers to assure target gene introgression, and heterozygous individuals were selected for the next-round backcross. The same strategy was followed for the next two rounds of backcrossing. BC_3_F_1_ lines for 1401, 1413, 1434, and 1445 were obtained. Individuals of BC_3_F_1_ lines were subject to traditional selection by breeders for agronomic traits including taste and southern rust resistance. Individuals with poor field performance and bad taste were discarded. For 1401, an additional round of backcrossing was adopted to ensure the palatability of the introgressed line. Selected lines were self-pollinated to produce BC_3_F_2_ or BC_4_F_2_. Southern rust resistance and agronomic performance were examined at BC_3_F_2_/BC_4_F_2_ during the autumn season of 2019; background selection was also conducted at this generation.

### Background selection

BC_3_F_2_ or BC_4_F_2_ lines resistant to southern rust and displaying similar agronomic performance to the original parent lines were analyzed using a GoldenGate 6 K SNP chip (Illumina, San Diego, CA, USA) as described previously (Li et al. 2017). Percentage recovery of the genomic background was calculated as RPG = [(*X* + 1 / 2*Y*) × 100] / *N*, where *N* is the total number of parental polymorphic markers screened, *X* is the number of homozygous alleles matching the recurrent parent, and *Y* is the number of heterozygous alleles. Individuals displaying the highest genome recovery rate were selected and selfed twice to generate converted lines, namely BC_3_F_4_ lines for 1413, 1434, and 1445, and BC_4_F_4_ lines for 1401. Newly developed converted lines were designated 1401R, 1413R, 1434R, and 1445R.

### Evaluation of southern rust resistance

Disease severity under natural inoculation was scored and evaluated by rating on a 1 to 9 scale, 22 days after pollination in November (Lu et al. 2020), when the temperature and moisture are suitable for the breakout of southern rust annually. Plants were scored as highly resistant (1), resistant (3), moderately resistant (5), susceptible (7), or highly susceptible (9) according to the percentage of three ear leaves that were covered by orange spores. For inbred lines or hybrids, three replicates were grown and around 25 plants were scored in each replicate. Recipient and donor parents were grown as susceptible and resistant controls. Disease Severity Index (DSI) was used to represent southern rust severity. DSI was calculated as follows:

$$\mathrm{DSI}=\mathrm\Sigma\lbrack(\mathrm{score}\times\mathrm{number}\;\mathrm{of}\;\mathrm{plants}\;\mathrm{in}\;\mathrm{score})\;\times100/(9\times\mathrm{total}\;\mathrm{number}\;\mathrm{of}\;\mathrm{plants})\rbrack$$

### Agronomic performance and biochemical analysis

Converted lines were crossed with the original male parents to reconstitute hybrids. A field plot was planted in a randomized complete block design. Plants were grown in 2 rows, and each row was 3 m long, with row-to-row and plant-to-plant distances of 65 cm and 28 cm, respectively. Agronomic performance of the converted inbred lines and hybrids along with their original parent lines was calculated. For inbred lines, agronomic trait evaluation comprised days to 50% anthesis, plant height, ear weight, ear height, cob length, cob width, number of rows, and number of kernels per row. For the hybrids, the additional traits of ear weight, seed length, seed width, and seed thickness were evaluated. Given that sugar content is of great importance to sweet corn taste, fresh ears were harvested 22 days after pollination, and 100 seeds from the middle part of each ear were collected for biochemical analysis. Contents of glucose, fructose, and sucrose were estimated using a high-performance liquid chromatography system as described by Abbas et al. (2020). Data were subjected to analysis of variance (ANOVA) to determine significant differences between lines or hybrids using SPSS software (https://www.ibm.com/cn-zh/products/spss-statistics).

## Results

### Identification of polymorphic markers

*RppQ* is located on the short arm of chromosome 10 between the SCAR marker MA7 and the AFLP marker M-CCG/E-AGA157 with distances of 0.46 cm and 1.71 cm (Zhou et al. 2007). A sequence of bin 10.01 (Chr10: 2,650,397 ~ 5,008,368) encompassing the *RppQ* mapping region was downloaded from the B73 release genomic annotation database for marker development. We tested 96 markers in a polymorphism survey; five markers were found to be polymorphic in the target region, and at least two of these showed polymorphisms between the donor and the four recurrent parental inbred lines. The newly developed markers were named M0607, M0801, M0903, M3301, and M3402; primer sequences are listed in Table S1. The type of polymorphism between Qi319 and the four sweet corn lines is shown in Table S2. Seedlings were genotyped using the corresponding markers for foreground selection to identify heterozygous individuals in the population carrying the *RppQ* fragment from the donor parent. M0904 and M3402 were used for foreground selection in 1401/Qi319 populations. M0801, M0904, M3301, and M3402 were used for 1413/Qi319 populations. M0607 and M0801 were used for 1434/Qi319 populations. M0904 and M3301 were used for 1445/Qi319 populations.

### Marker-assisted introgression of RppQ

To introgress the *RppQ* gene into 1401, 1413, 1434, and 1445, the donor inbred Qi319 was hybridized with four sweet corn lines during the 2016 autumn season, and five ears were harvested from each cross. In the spring of 2017, F_1_ seeds were planted and identified using a co-dominant marker to assure hybridity and further backcrossed with the respective recurrent parents to generate BC_1_F_1_ lines. BC_1_F_1_ lines were planted in the next season (autumn 2017) and screened using foreground selection markers. Individuals identified as having introgression of *RppQ* were selected for the next-round backcross. During 2017–2018, three rounds of backcrossing were carried out. We obtained 153, 138, 140, and 135 BC_3_F_1_ ears for 1401, 1413, 1434, and 1445, respectively. We did not calculate the effect of southern rust resistance improvement due to *RppQ* introgression during the whole backcross procedure.

We planted 153, 138, 140, and 135 BC_3_F_1_ ear rows of converted lines together with their original lines in the autumn of 2019. Those displaying low germination rate (< 85%) were eliminated. Plant height, ear height, flowering time, and plant architecture were visually assessed at flowering stage, and lines that were distinct from the original lines were excluded. Ears were harvested 22 days after pollination. Ear traits including ear length, ear width, number of kernel rows per ear, and number of kernels per row were calculated, and introgressed lines displaying similar traits to the original lines were saved. We also selected for ear appearance including silk and kernel color, and lines with shorter husk extension that did not cover above the ear tip were eliminated. A direct taste test of fresh ears was also conducted by breeders, and those ears with thick pericarp and bad taste were ruled out. After rigorous selection, we saved 18, 20, and 16 individuals with the desired traits for 1413, 1434, and 1445, respectively, and these were self-crossed to generate BC_3_F_2_ lines. One more generation of backcrossing was performed for line 1401 to produce a sweet corn with better taste, and 23 BC_4_F_1_ lines were finally self-crossed to produce BC_4_F_2_ lines. Individuals with the donor-type allele, homologous for *RppQ*, were selected according to a theoretical genetic segregation ratio of 1:2:1. The BC_3_F_2_ or BC_4_F_2_ lines were also screen for phenotypic traits, including agronomic performance and southern rust resistance. After several rounds of filtering, four individuals from each recurrent parent were selected for background selection.

### Background selection of introgressed lines

**Fig. 2:**
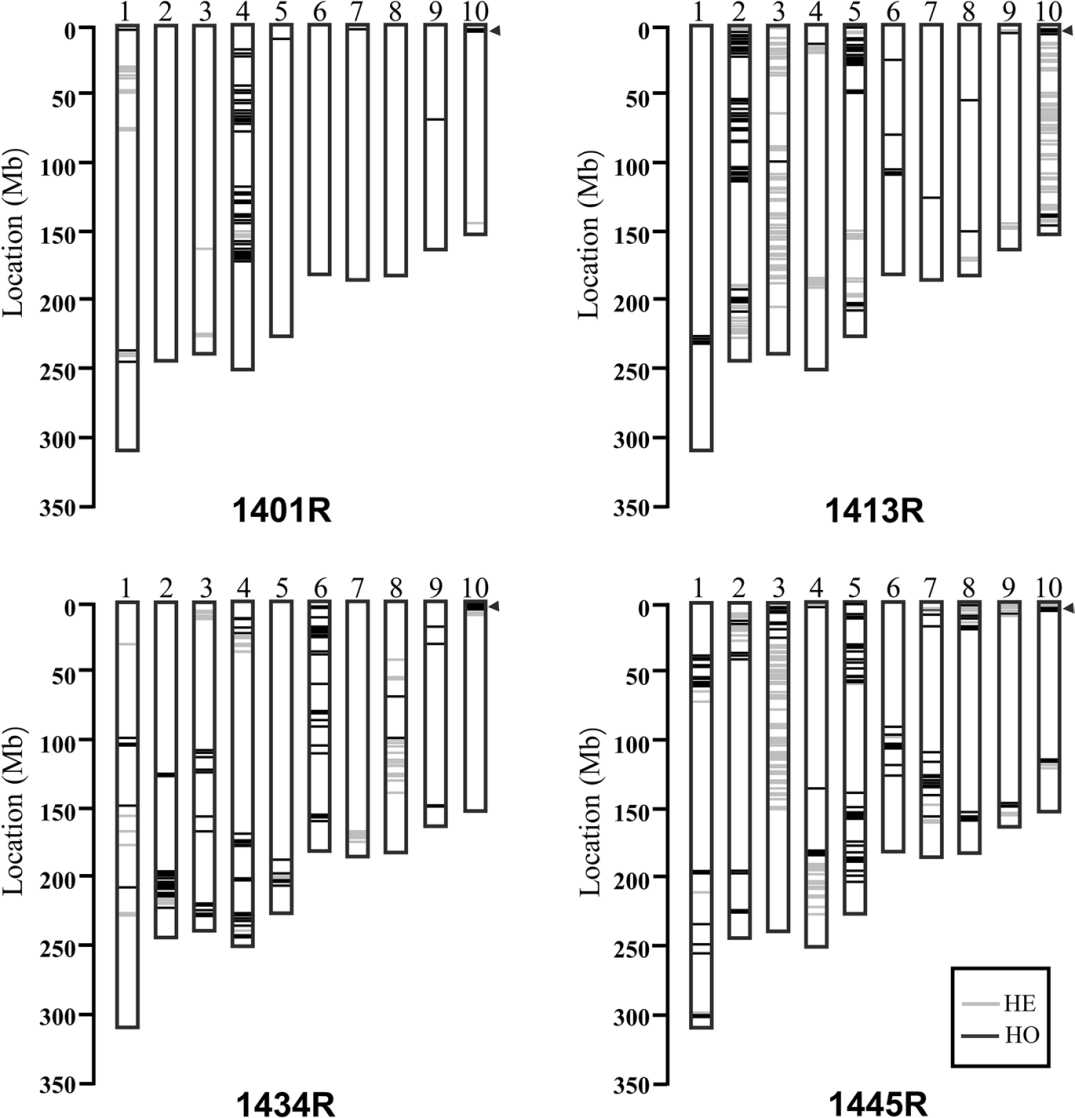
Genetic background and southern rust disease severity of introgressed lines. Genetic background of four converted lines determined using a 6 K SNP chip. *HE* heterozygous genotype from Qi319 represented by a gray line, *HO* homozygous genotype from Qi319 represented by a black line. Black triangles represent the positions of *RppQ*

To assess the genetic background of the converted lines, four individuals from each recurrent parent that displayed similar morphological phenotypes to the recurrent parents were selected for a genome-wide 6 K SNP chip scan (Table S3). Lines possessing the greatest genome recovery rate, up 97.9%, 92.6%, 93.8%, and 92.3% of the genomes of 1401, 1413, 1434, and 1445, respectively, as measured by the percentages of polymorphic markers showing the same genotype as the recurrent parent, were designated converted lines (Fig. 2). *RppQ* from donor Qi319 was introduced into the four sweet corn lines via introgression of fragments of approximately 894 kb, 4720 kb, 7460 kb, and 2763 kb, respectively. Newly developed lines were named 1401R, 1413R, 1434R, and 1445R. The chosen lines displayed similar agronomic traits to the original lines (Table 1) and were self-crossed for another two generations during the next two growing seasons or crossed with the original parental sweet corn lines to produce hybrids in the BC_3_F_3_/BC_4_F_3_ generation. Seeds were harvested and bulked for follow-up selection of agronomic traits and southern rust resistance testing.

**Table 1:** Comparison of agronomic performance between the converted and original lines

Inbred line	Plant height (cm)	Ear height (cm)	Days to 50% anthesis	Days to 50% silking	Cob length (cm)	Cob girth (cm)	Number of kernel rows per ear	Number of kernels per row
1401	190.04 ± 16.44	76.26 ± 11.76	53.70 ± 0.95	58.15 ± 0.66	14.28 ± 1.57	3.97 ± 0.37	15.04 ± 1.87	27.33 ± 3.37
1401R	187.85 ± 11.71	72.77 ± 13.51	53.67 ± 0.94	58.67 ± 0.47*	13.81 ± 1.28	3.91 ± 0.26	14.92 ± 2.43	25.23 ± 4.06
*P* value	0.670	0.408	0.921	0.039	0.393	0.584	0.862	0.123
1413	132.93 ± 10.43	55.84 ± 6.71	47.00 ± 0.68	50.10 ± 0.88	11.00 ± 1.42	3.56 ± 0.33	13.61 ± 1.50	24.35 ± 3.21
1413R	127.18 ± 7.73	55.20 ± 8.35	47.27 ± 0.59	50.33 ± 0.49	11.21 ± 0.84	3.61 ± 0.24	14.40 ± 1.35	22.47 ± 5.68
*P* value	0.108	0.986	0.203	0.508	0.600	0.182	0.092	0.156
1434	144.33 ± 12.87	48.15 ± 10.78	48.00 ± 0.30	51.92 ± 1.04	11.96 ± 1.85	3.49 ± 0.49	13.62 ± 1.36	21.92 ± 3.26
1434R	148.75 ± 6.84	53.43 ± 7.99	49.50 ± 1.07**	51.63 ± 0.52	10.58 ± 2.98	3.33 ± 0.33	13.93 ± 1.77	20.71 ± 4.75
*P* value	0.239	0.150	0.001	0.461	0.164	0.316	0.619	0.390
1445	140.67 ± 8.30	43.39 ± 10.27	47.33 ± 1.03	52.44 ± 2.62	11.51 ± 2.50	3.62 ± 0.42	13.33 ± 1.68	19.31 ± 4.67
1445R	145.78 ± 9.39	49.42 ± 14.36	46.67 ± 0.49	50.67 ± 0.49	10.13 ± 2.00	3.64 ± 0.44	13.17 ± 2.76	18.64 ± 4.86
*P* value	0.244	0.140	0.470	0.280	0.122	0.884	0.838	0.708

^*^P ≤ 0.05, significant differences between converted and original lines; **P ≤ 0.01, significant differences between converted and original lines

### Converted lines and derived hybrids show enhanced resistance to southern rust

**Fig. 3:**
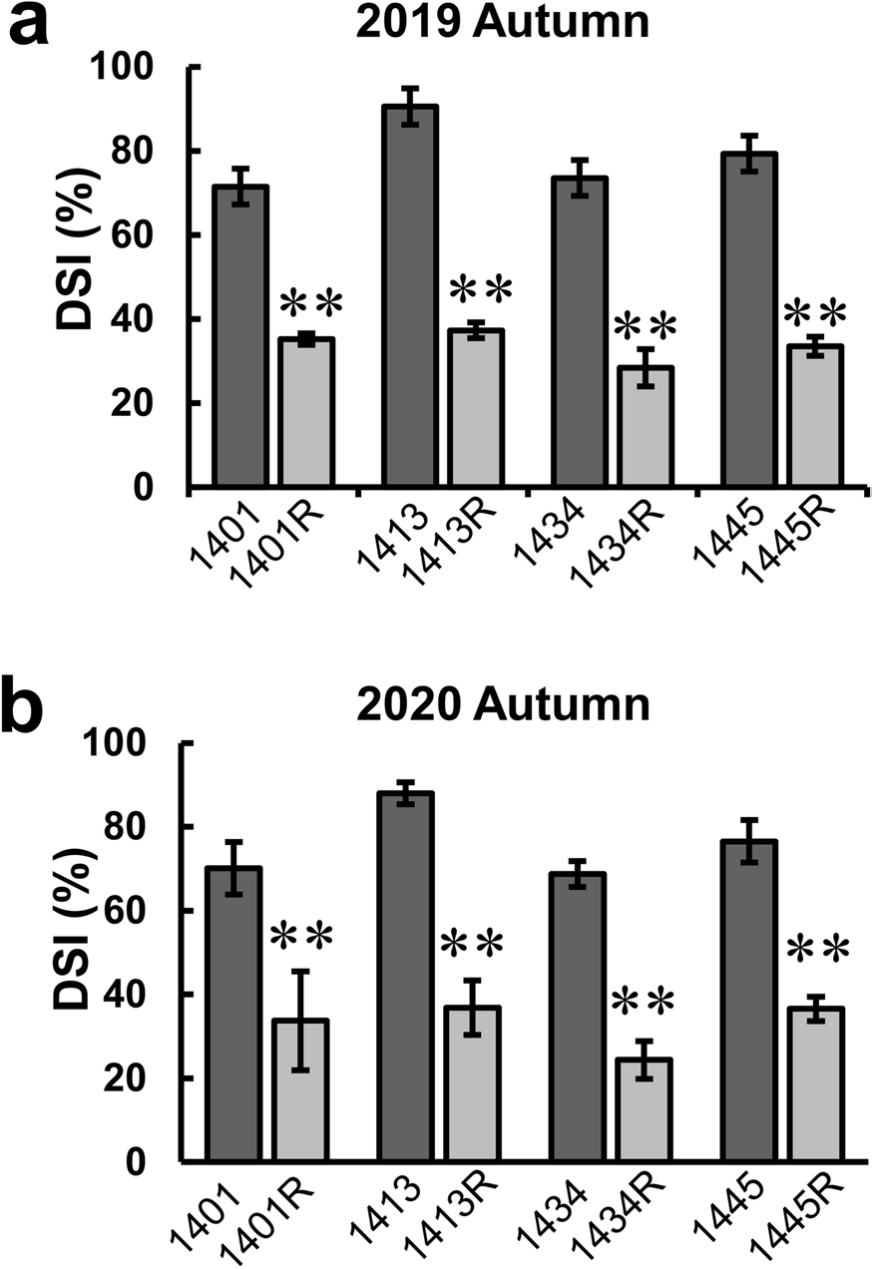
DSIs of converted lines and the respective original lines. **a** DSIs of different lines in the autumn season of 2019. **b** DSIs of different lines in the autumn season of 2020. **P ≤ 0.01, significant differences between converted and original lines

Converted and original lines were exposed to natural conditions of southern rust in the autumn seasons of 2019 and 2020. All of the converted lines displayed significantly enhanced resistance compared with non-converted lines, with DSI varying from 28.42 to 38.33% compared with 71.48 to 90.52% for the non-converted lines in the autumn season of 2019 (Fig. 3a). Similarly, converted lines 1401R, 1413R, 1434R, and 1445R displayed lower DSI (24.43 to 36.58%) than the original lines (68.74 to 88.01%) in the autumn season of 2020 (Fig. 3b), demonstrating successful improvement of southern rust resistance through *RppQ* introgression.

**Fig. 4:**
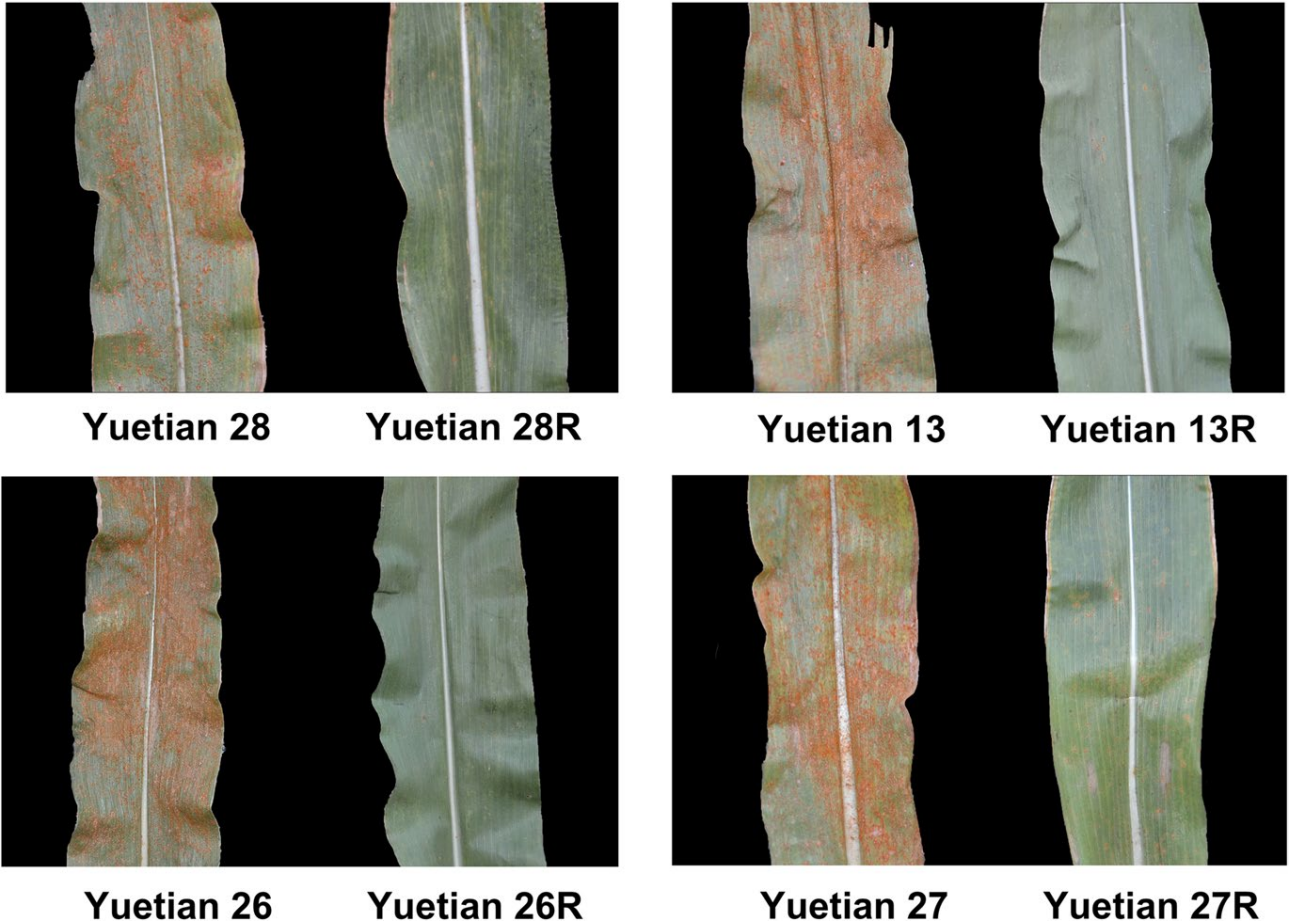
Improvement of southern rust resistance in converted hybrids. Leaf appearance of original and reconstituted hybrids under natural inoculation with *R. polysora*

**Fig. 5:**
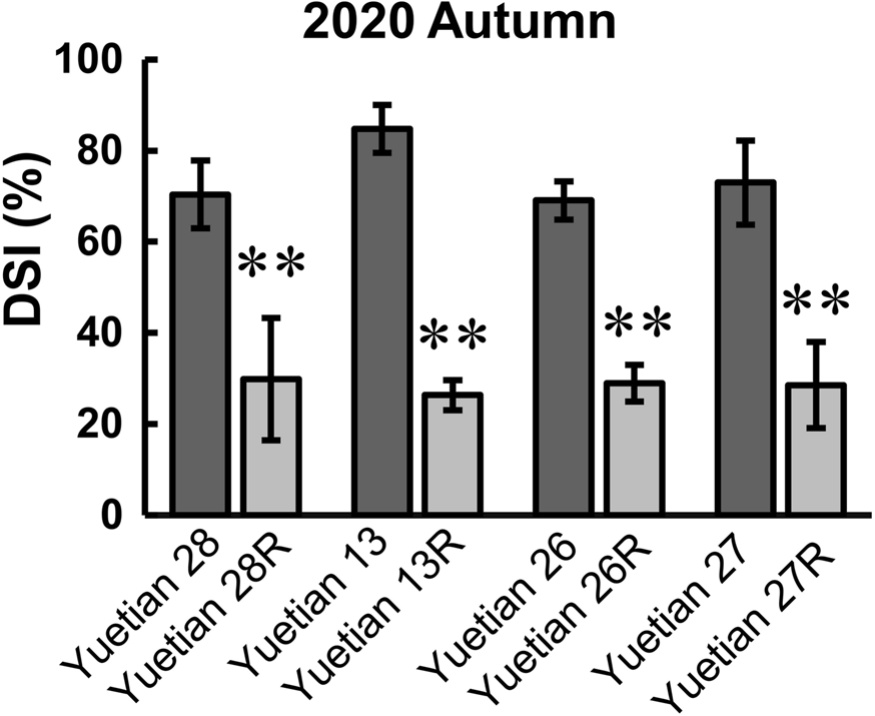
DSIs of newly derived hybrids and respective original hybrids in the autumn season of 2020. **P ≤ 0.01, significant differences between converted and original lines

Converted lines were crossed with the original parental sweet corn lines 13F, 26F, 27F, and 28F to construct newly converted hybrids. The derived hybrids, ‘Yuetian 13R,’ ‘Yuetian 26R,’ ‘Yuetian 27R,’ and ‘Yuetian 28R,’ were also evaluated for southern rust resistance, compared with the original hybrids ‘Yuetian 13,’ ‘Yuetian 26,’ ‘Yuetian 27,’ and ‘Yuetian28.’ The results presented in Fig. 4 show the resistance of sweet corn hybrids to southern rust. Hybrids with or without the *RppQ* gene differed significantly in field tests. DSI of the converted hybrids ranged from 26.36 to 29.84%, while that of the original hybrids ranged from 69.06 to 84.75%, suggesting that southern rust resistance was improved successfully after introgression of the *RppQ* through marker-assisted selection (MAS) (Fig. 5).

### Agronomic traits and sugar content of derived hybrids

Agronomic traits including plant height, ear height, fresh ear weight, cob weight, cob length, cob width, number of kernel rows per ear, and number of kernels per row were calculated from field trials in 2020 (Table 2). Morphological characteristics of the reconstituted hybrids exhibited a high resemblance to those of the respective original hybrids. Considering that sweetness is of great importance for consumer satisfaction, we compared the content of glucose, fructose, and sucrose from the converted hybrids with those of the non-converted hybrids. As shown in Table 3, sugar content of the converted hybrids was comparable to that of the respective original, with the difference between pairs being insignificant. The levels of sucrose (226.95 to 369.36 mg/g), glucose (34.74 to 63.98 mg/g), and fructose (18.19 to 34.73 mg/g) in the original hybrids were similar to the levels of sucrose (254.03 to 378.50 mg/g), glucose (39.21to 69.14 mg/g), and fructose (19.44 to 32.08 mg/g) in the derived hybrids, respectively, suggesting that introgression of the *RppQ* fragment did not affect the sugar content of sweet corn.

**Table 2:** Comparison of agronomic performance between the reconstituted and original hybrids

Hybrid	Plant height (cm)	Ear height (cm)	Days to 50% anthesis	Days to 50% silking	Cob length (cm)	Cob width (cm)	Fresh ear weight (g)	Cob weight (g)	Number of kernel rows per ear	Number of kernels per row
Yuetian 13	174.5 ± 6.81	69.75 ± 2.5	47.5 ± 0.58	50.5 ± 0.54	4.97 ± 0.25	4.97 ± 0.25	325.7 ± 30.93	249.5 ± 9.74	18.0 ± 1.63	34.5 ± 2.38
Yuetian 13R	179.4 ± 3.64	71.8 ± 2.86	48.2 ± 0.84	51.2 ± 0.84	4.92 ± 0.19	4.92 ± 0.19	330.4 ± 23.60	240 ± 15.61	17.6 ± 0.89	35.6 ± 3.65
*P* value	0.206	0.297	0.200	0.200	0.785	0.719	0.805	0.325	0.652	0.621
Yuetian 26	186.4 ± 6.04	75.0 ± 5.24	50.2 ± 0.84	53.4 ± 0.89	5.40 ± 0.12	5.40 ± 0.12	421.8 ± 16.34	314.8 ± 17.25	18.4 ± 1.67	36.6 ± 2.19
Yuetian 26R	191.8 ± 9.20	77.2 ± 6.14	49.6 ± 0.89	53.0 ± 0.71	5.42 ± 0.08	5.42 ± 0.08	417.5 ± 31.30	317.2 ± 23.7	18.8 ± 1.09	39.4 ± 3.13
*P* value	0.273	0.559	0.305	0.455	0.127	0.771	0.778	0.859	0.667	0.140
Yuetian 27	175.6 ± 3.51	70.4 ± 6.15	47.6 ± 0.54	51.4 ± 0.55	4.42 ± 0.18	4.42 ± 0.18	306.1 ± 13.70	258.2 ± 10.60	15.6 ± 0.91	34.8 ± 1.48
Yuetian 27R	179.6 ± 7.26	73.4 ± 7.76	47.0 ± 0.31	51.2 ± 0.73	4.41 ± 0.16	4.41 ± 0.16	304.4 ± 17.30	255.4 ± 16.51	15.2 ± 1.09	34.0 ± 2.24
*P* value	0.301	0.517	0.172	0.667	0.486	0.856	0.875	0.774	0.545	0.524
Yuetian 28	222.2 ± 7.36	95.6 ± 6.34	55.4 ± 0.54	58.6 ± 0.58	5.24 ± 0.16	5.24 ± 0.16	417.2 ± 22.78	303.0 ± 23.05	18.1 ± 1.41	38.8 ± 2.17
Yuetian 28R	224.8 ± 5.89	99.4 ± 3.51	54.8 ± 0.84	58.2 ± 1.31	5.20 ± 0.12	5.20 ± 0.12	413.6 ± 24.88	298.6 ± 13.48	17.6 ± 2.61	40.2 ± 2.49
*P* value	0.555	0.275	0.217	0.486	0.311	0.678	0.817	0.619	0.694	0.371

**Table 3:** Comparison of sugar content the between reconstituted and original hybrids

Hybrid	Fructose (mg/g)	Glucose (mg/g)	Sucrose (mg/g)
Yuetian 13	18.19 ± 2.16	34.74 ± 6.91	226.95 ± 49.35
Yuetian 13R	19.44 ± 1.89	39.21 ± 3.49	254.03 ± 31.76
*P* value	0.491	0.373	0.496
Yuetian 26	34.73 ± 2.99	58.42 ± 4.95	369.36 ± 61.00
Yuetian 26R	29.56 ± 5.98	57.35 ± 9.95	331.77 ± 40.91
*P* value	0.252	0.876	0.425
Yuetian 27	31.62 ± 3.46	63.98 ± 10.73	378.50 ± 71.80
Yuetian 27R	32.08 ± 7.09	69.14 ± 20.62	292.45 ± 30.66
*P* value	0.923	0.721	0.129
Yuetian 28	29.03 ± 4.02	62.95 ± 9.04	362.00 ± 37.65
Yuetian 28R	29.77 ± 5.51	62.81 ± 3.29	349.67 ± 16.37
*P* value	0.862	0.921	0.630

## Discussion

Sweet corn is the sweet version of maize, a result of recessive mutations in genes controlling sugar metabolism. However, its yield and quality are severely affected by southern rust. Airborne spores with the ability to travel long distances and the complexity of pathogen races make controlling this pathogen difficult. Unlike traditional field corn, the kernels of sweet corn are delicious and tender and are consumed directly as a vegetable. Thus, chemical control of southern rust is not a preferred method. Developing and improving sweet corn varieties harboring disease resistance is the most economical and reliable way to resolve the problem. Despite years of field trial experience, temperate sweet corn is short of highly resistant lines. We therefore turned to Qi319, a field corn inbred line possessing the southern rust resistance gene *RppQ*, for our marker-assisted breeding program, hoping to create sweet corn germplasm with enhanced rust resistance. In this study, we transferred *RppQ* accurately into four elite sweet corn lines, 1401, 1413, 1434, and 1445, using a MAS approach. This resulted in four new lines resistant to southern rust: 1401R, 1413R, 1434R, and 1445R. These converted lines and their derived hybrids were thoroughly evaluated for agronomic traits, particularly sweetness. As expected, the new hybrids with improved southern rust resistance were developed without losing the desirable traits of the original hybrids.

Conventional sweet corn breeding has focused on sweetness, kernel color, germination rate, husk coverage, flavor, pericarp thickness, etc., traits not typically the target for field corn breeding. One-to-one genome alignment between sweet corn line Ia453-sh2 and six field corn accessions resulted in an average synteny calculation of 68.95%, with little overlap of genomic regions under selection between sweet corn and field corn (Hu et al. 2021). The narrow genetic base has always been a major constraint for sweet corn breeding. Collaboration between sweet and field corn research provides an opportunity for using field corn with desirable features for breeding, thereby enriching the genetic base of sweet corn. Field corn with identified resistance genes offers a plausible germplasm resource for resistance improvement; however, this resource is seldom utilized by sweet corn breeders because they are worried about introducing undesirable features such as poor husk cover, poor eating quality, tough pericarp, and lack of tenderness and flavor. Our breeding program has provided a successful path for using marker-assisted resistance gene introgression from field corn into sweet corn through careful scrutiny of agronomic traits. Besides screening for southern rust resistance, our selection process paid great attention to eliminating side effects caused by introducing field corn genetic material. Furthermore, taste tests by experienced breeders were decisive in the final selection of the backcross population. As a result, agronomic traits and sweetness did not exhibit significant differences between the original and converted hybrids, indicating that important traits were recovered after marker-assisted backcrossing. With *RppQ* successfully introduced into the sweet corn genetic pool, our converted lines can be used as a source of southern rust resistance to improve other parental lines. A total of 186 functional genes or major QTLs of maize have been mapped or cloned, and information on molecular markers and donor parents has been summarized (Ma et al. 2019). This will enable easy application of molecular markers for creating elite sweet corn lines with great eating quality and superior resistance to disease, which will be of great importance to sweet corn breeding.

*RppQ* has been known as a resistance gene for nearly 20 years; however, the relationship between *RppQ* and the racial specificity of southern rust has not yet been determined. Although Qi319 has conferred a long period of effective resistance, we still worry about the rapid evolution of *P. polysora* to overcome this resistance. *Rpp9*, which was successfully used in the USA for 20 years, was overcome by a new race of *P. polysora* (Dolezal, et al. 2009). A novel major QTL, *qSCR6.01*, detected on chromosome 6, was fine mapped from Qi319, explaining 17.99 ~ 24.15% of the total phenotypic variation under different environments (Lu et al. 2020). This will enable pyramiding of two resistance loci in one resistant line. We hope that a quantitative gene conferring stable and durable resistance against southern rust will be cloned and used for molecular-assisted breeding in the future.

## Supplementary Information

Below is the link to the electronic supplementary material.

Supplementary file1 (DOCX 18 KB)

## Data Availability

Not applicable.
