# Supplementary material for: Introgression of the RppQ gene from field corn improves southern rust resistance in sweet corn
Source: Mol Breed. 2022 Aug 30;42(9):53. doi: 10.1007/s11032-022-01315-7 (PMC10248694; doi:10.1007/s11032-022-01315-7)
Supplement: Supplementary file 1 — Supplementary file1 (DOCX 18 KB) [file 11032_2022_1315_MOESM1_ESM.docx]

**Table S1** List of markers used for foreground selection

| Marker | Sequence (5′-3′) | Size in donor parent (bp) | Position in B37  (Chr. 10) |
| --- | --- | --- | --- |
| M0607 | FP: GATTTGCATGGGACCAGGAC  RP: TGTTGCGACGCTTCCATATG | 695 | 4,019,385-4,020,079 |
| M0801 | FP: AGCTCGATCAATCCAGGGAG  RP: GATACCGTGGTTAGAGGCGA | 639 | 4,064,249-4,064,893 |
| M0904 | FP: CACAAGCAGGATGGTAGCAC  RP: TCCTCCAGAATCCAGATGCA | 537 | 4,095,946-4,096,482 |
| M3301 | FP: ATGTGCATGTGGTGGTGAAC  RP: ACTATGGCATGGATCGCTCA | 841 | 4,661,660- 4,660,777 |
| M3402 | FP: CTGCTATGACAAGTGCCGAC  RP: CCTCTGCCTAATGAAACGCC | 393 | 4,672,567-4,672,751 |

FP, forward primer; RP, reverse primer

**Table S2** Polymorphisms of five markers in the *RppQ* region between the donor parent Qi319 and four recurrent parents

| Marker | Recurrent parent | | | |
| --- | --- | --- | --- | --- |
|  | 1401 | 1413 | 1434 | 1445 |
| M0607 | - | - | 2 | - |
| M0801 | - | 2 | 1 | - |
| M0904 | 1 | 1 | - | 1 |
| M3301 | - | 2 | - | 2 |
| M3402 | 2 | 1 | - | - |

1, Dominant marker; 2, co-dominant marker; -, no difference in fragment size between donor and recurrent parent

**Table S3** Distribution of SNP markers across 10 maize chromosomes for background selection

| Chr. | Recurrent parent | | | |
| --- | --- | --- | --- | --- |
|  | 1401 | 1413 | 1434 | 1445 |
| Chr.1 | 389 | 376 | 329 | 382 |
| Chr.2 | 328 | 291 | 242 | 281 |
| Chr.3 | 306 | 265 | 252 | 292 |
| Chr.4 | 291 | 264 | 248 | 271 |
| Chr.5 | 275 | 267 | 267 | 278 |
| Chr.6 | 225 | 236 | 164 | 212 |
| Chr.7 | 211 | 229 | 210 | 207 |
| Chr.8 | 244 | 227 | 177 | 220 |
| Chr.9 | 199 | 207 | 177 | 213 |
| Chr.10 | 163 | 180 | 163 | 183 |
| Total | 2631 | 2542 | 2229 | 2539 |
